# Dietary Pattern and Dietary Energy from Fat Associated with Sarcopenia in Community-Dwelling Older Chinese People: A Cross-Sectional Study in Three Regions of China

**DOI:** 10.3390/nu12123689

**Published:** 2020-11-30

**Authors:** Cheng Li, Bingxian Kang, Ting Zhang, Hongru Gu, Pengkun Song, Jingyi Chen, Xile Wang, Bin Xu, Wenhua Zhao, Jian Zhang

**Affiliations:** 1 National Institute for Nutrition and Health, Chinese Center for Disease Control and Prevention, 27 Nanwei Road, Xicheng District, Beijing 100050, China; lichengyys@126.com (C.L.); songpk@ninh.chinacdc.cn (P.S.); jingyich@126.com (J.C.); zhaowh@chinacdc.cn (W.Z.); 2 Wuyuan County Center for Disease Control and Prevention, 105 Shiji Road, Wuyuan 015100, China; kbingxian@163.com (B.K.); wyxjkzxwxl@126.com (X.W.); 3 Yuexiu District Center for Disease Control and Prevention, 23 Jiaochang West Road, Guangzhou 510030, China; tingzhangyx@yeah.net (T.Z.); xubin6710@163.com (B.X.); 4 Taicang City Center for Disease Control and Prevention, 36 Xianfu West Street, Taicang 215400, China; bcghr@126.com

**Keywords:** dietary pattern, dietary fat, sarcopenia, community-dwelling older Chinese

## Abstract

Associations between dietary patterns (DPs) and sarcopenia remain controversial, and fewer studies have mentioned the relationship between dietary energy composition and sarcopenia. The present cross-sectional study was conducted in three regions of China, to detect the associations between DPs and sarcopenia, and to identify the influencing nutrients. Exploratory factor analysis was conducted for DP identification. Logistic regressions were performed to explore the associations between DPs and sarcopenia. Dietary nutrients and dietary energy composition were calculated and compared among different DPs. Three DPs were identified from 861 community-dwelling older people. The “mushrooms–fruits–milk” pattern was negatively associated with sarcopenia (*OR* = 0.33, 95% *CI* = 0.14~0.77, *p*-trend = 0.009). Subjects in the highest quartile of the “mushrooms–fruits–milk” pattern showed more abundant intake (1.7 g/kg/d) of dietary protein, and lower percentage (31%) of energy from fat (PEF) than the other two DPs. Further analyses indicated that lower PEF (<30%) was negatively associated with sarcopenia. In conclusion, the “mushrooms–fruits–milk” pattern was negatively associated with sarcopenia in community-dwelling older Chinese people. This pattern showed abundant protein intake and low PEF, which may partially contribute to its protective effect on sarcopenia. Therefore, besides protein, dietary fat and PEF may also be considered in the prevention and management of sarcopenia.

## 1. Introduction

Sarcopenia, characterized by muscle mass decline and muscle quality impairment, has caused widespread concern in recent years [1]. It is significantly associated with physical capability decline, disability, and increased mortality in older people [2]. Aging, diet, physical activity, metabolic balance, and inflammation were proven to be associated with sarcopenia in previous studies [2,3,4,5]. As a modifiable factor among those influencing factors in older people, diet was considered in the prevention and management of sarcopenia [3,4].

Due to the complex etiologies of diseases, single food or nutrient research may be difficult to explain due to the multiple relationships between diet and health outcomes [6]. Compared with the traditional analysis of single food or nutrient, dietary pattern (DP) analysis has been identified as a more comprehensive method to characterize diet. Previous studies indicated that, DP analysis could offer a broader picture of food consumption, and reflect a more real dietary habit [7]. Furthermore, with the comprehensive reflect of diet and the superiority of reducing the collinearity between various food groups, DP analysis could provide some practical approaches to disease prevention [4,6,7]. Therefore, DP analysis has been progressively used by researchers to explore the relationships between diet and health outcomes [7,8,9,10]. Recently, several studies discussed the associations between DP and sarcopenia [3,4]. A cross-sectional study in Italy found the Mediterranean-style pattern was positively associated with physical performance in older people [11]. Another cohort study in Finland found the Nordic diet pattern, with favorable food of fruits, berries, vegetables, cereals, low-fat milk, and fish, was significantly associated with better gait speed and skeletal muscle strength in older women [12]. A cohort study in the UK found that, keeping a “healthy” DP in adult lifetime, characterized by higher consumption of fresh fruits, leafy vegetables, and wholegrain bread, may increase physical performance in older age [13]. While, most of those studies were conducted in a particular region [4], the identified DPs with regional characteristics were limited to extrapolate in other regions or populations. Further, most of the previous studies only discussed the associations between DPs and sarcopenia, the potentially influencing nutrients and dietary energy composition were rarely mentioned.

Compared with DP studies, more studies discussed the associations between dietary nutrients and sarcopenia [4]. Protein has been recognized as one of the most important nutrients in the regulation of muscle health in older people [14,15]. Reasonable protein intake could stimulate muscle protein synthesis, and maintain skeletal muscle mass and muscle function [16]. However, the associations between dietary nutrients and sarcopenia are rather complex [4,17]. In addition to dietary protein, dietary fat, branched-chain amino acids, calcium, selenium, and magnesium, have been involved in muscle cell metabolism by different regulatory pathways, including muscle protein synthesis, muscle fiber regulation, antioxidant stress, and muscle cell atrophy [16,18,19,20,21]. Separating the influence of one kind of nutrients or dietary components from the whole diet, may not be enough to explain the relationship between diet and sarcopenia [4].

Based on those previous studies, we hypothesized that: DPs may associate with sarcopenia in community-dwelling older people. Furthermore, in addition to dietary protein, there may be other nutrients involved in the development of sarcopenia. Therefore, the present cross-sectional study was conducted to explore the associations between DPs, dietary nutrients, and sarcopenia in community-dwelling older Chinese people. In the present study, diet and muscle condition of community-dwelling older people from three regions of China (Wuyuan, Taicang, and Yuexiu, located in the north, east, and south of China, respectively) was investigated. According to the eigenvalue of food intake, dominant DPs were extracted by exploratory factor analysis. The relationship between DPs and sarcopenia was discussed to find the DP with potential protective effect on sarcopenia. Moreover, the intake of dietary nutrients that participated in muscle metabolism (including protein, fats, minerals, branched-chain amino acids, and dietary energy composition) was calculated, and the variation of nutrients among DPs was analyzed comprehensively to further understand the different effects of DPs on sarcopenia.

## 2. Materials and Methods

### 2.1. Participants and Study Design

This cross-sectional study was conducted in three demographically representative regions of China in 2018. Dietary habits and food supply in the three regions are significantly different. The main staple food in Wuyuan is wheat. Taicang is located by the Yangtze River and there are more aquatic products than Wuyuan. Yuexiu located in the Pearl River Delta, and has more variety in the supply of vegetables and fruits than the other two regions. About 300 community-dwelling older people aged 65 years old or older were respectively recruited from two to four randomly selected local communities in each region. Individuals with the following conditions were excluded: (1) disability for daily activity or physical measurement; (2) refuse for body composition analysis; (3) pacemaker or severe edema; (4) obstacle for communication or investigation; and (5) under 65 years old. All of the participants signed an informed consent before the enrollment. There were 861 older subjects that were finally analyzed in the present study. This study was conducted in accordance with the Declaration of Helsinki, and was approved by the Ethics Committee of National Institute for Nutrition and Health (NINH), Chinese Center for Disease Control and Prevention (CCDC), with the ethics number of 2018-014.

### 2.2. Questionnaire and Dietary Assignment

Demographics, lifestyle, physical activity, and diagnosis history of relevant diseases were assessed and collected by trained public health investigators from the local Center for Disease Control and Prevention (CDC) and hospital. Age, gender, and regional information in health file of the participant were collected at the beginning of the study, and were rechecked by investigator in the face-to-face interview. An international physical activity questionnaire was used to collect the information of exercise activity [22]. Total metabolic equivalent (MET) value was calculated from the frequency (week), duration (minutes) and type of exercise activity (MET). Total MET < 600 MET-min/w was recognized as low exercise activity, 600 MET-min/w~3000 MET-min/w was recognized as moderate exercise activity, total MET > 3000-min/w was recognized as high exercise activity. Lifestyle and smoke information were recorded in the questionnaire by investigator in the face-to-face interview. Hypertension, type 2 diabetes (T2D), and cardiovascular disease were identified as non-communicable chronic diseases (NCDs) in the present study, diagnosis history, and treatment information of those NCDs were assessed by the investigator and recorded in the questionnaire. A 64-item food frequency questionnaire (FFQ), which represented a slightly expanded version of the verified FFQ that was used in China National Nutrition and Health Surveys (CNNHS) [23,24]. The investigator asked and recorded the usual intake amount and frequency of each food item over the past year. The intake amount and the portion size were explained to each participant by the standard catalog of pictures of each kind of food, which was edited by NINH of CCDC and used in CNNHS. The daily intake amount of each item of food was calculated according to the food intake amount and intake frequency. Daily intake amounts of dietary energy, dietary nutrients related to muscle health were respectively calculated using the Chinese Food Composition Table [25]. There were 64 items of food that were then merged into 21 food groups for DP analysis and dietary variety (DV) score calculation.

### 2.3. Anthropometric Measurements

Body weight and height were measured once. Body mass index were calculated as body weight (kg)/height^2^ (m^2^). Mid-upper arm circumference (MUAC), calf circumference (CC), and waist circumference (WC) were measured twice by trained investigators, using a non-elastic tape. The average data of the two measurements of each circumference were used for the analysis. A bioimpedance device, in body 770 (Biospace, Seoul, Korea), were used to measure body composition, such as percentage of body fat mass (PBF), visceral fat area (VFA), fat-free mass (FFM), bone minerals content (BMC) and appendicular skeletal muscle mass (ASM) [26,27]. Skeletal muscle mass index (SMI) was calculated as ASM (kg)/height (m)^2^ and analyzed as the surrogate of skeletal muscle mass in this study [28]. Handgrip strength was assessed in a standing position with a grip strength meter (CAMRY EH101, Xiangshan, Zhongshan, China) [29] and performed the maximum strength twice with both hands. The maximum strength was analyzed as muscle strength in this study. The walk test was assessed twice by the time walking in a distance of 4 m at a habitual gait speed. The average speed was calculated and analyzed as gait speed in this study.

### 2.4. Diagnosis of Sarcopenia

Older subjects with low muscle mass and low grip strength or low gait speed could be diagnosed as sarcopenia [28,30]. This study was conducted in 2018, diagnosis criteria of sarcopenia recommended by the Asian Working Group for Sarcopenia in 2014 (AWGS2014) were used in the present study. In addition, the associations between DPs and sarcopenia defined by AWGS2019 were presented in Supplementary Table S4. According to the cut-off values of components of sarcopenia recommended by AWGS2014, low skeletal muscle mass: male with SMI < 7 kg/m^2^, or female with SMI < 5.7 kg/m^2^. Low grip strength: male with handgrip strength < 26 kg, or female < 18 kg. Low gait speed: a subject with gait speed < 0.8 m/s.

### 2.5. DV Score Calculation and DP Assessment

DV was scored by the variety section of Dietary Quality Index-Internal (DQI-I), which was calculated and verified in previous Chinese studies [31,32]. The DV score ranges from 0–20 and higher scores represent more various dietary compositions. Total variety score, overall food variety score, and protein source variety score were calculated as previous studies described [32]. In brief, overall food variety contained 5 major food groups: (1) meat, poultry, fish, and eggs; (2) dairy and beans; (3) grains; (4) fruits; and (5) vegetables. Overall, food variety score ranged from 0~15, and 3 points for 1 food group consumed daily. Protein source variety score contained 6 major protein sources: (1) meat; (2) poultry; (3) fish; (4) dairy; (5) beans; and (6) eggs. If there were more than 2 protein sources in daily diet, this component of food variety scored 5 points. If there were 2 protein sources in daily diet, this component scored 3 points. If there was only 1 protein source in daily diet, this component scored 1 point. No protein source scored 0 points.

Exploratory factor analysis was used to extract DPs based on the 21 food groups. Three factors were retained based on an eigenvalue bigger than 1.0 and the interpretability [33]. Each pattern was named with the food groups with the highest loading. Three DPs were identified and finally named “cereals–tubers–animal oils” pattern (DP1), “mushrooms-fruits-milk” pattern (DP2), and “animal foods” pattern (DP3). The factor score of each pattern was calculated for all of the subjects by summing the intake amounts of each food group and weighted by their factor loading. A higher factor score in the pattern represents greater conformity in this pattern.

### 2.6. Statistical Analysis

Quantitative data were presented as mean and standard deviance (SD), comparisons between two groups were performed by *t*-test or Wilcoxon test according to the examination of normal distribution. Categorical data were presented as number and percentage, comparisons between groups were performed by chi-square test. Partial correlation coefficients, with the adjustment of age, gender, and region, were calculated by Spearman rank correlation due to the non-normal distribution of food consumption in older people. A correlation matrix was carried out to reflect inter-relationships among all of the 21 food groups. Food groups were ranked by hierarchical clustering. Exploratory factor analysis was used for the identification of DPs. Partial correlations between DV score, DP score and muscle-related anthropometric characteristics were adjusted by age, gender, and region. Subjects were classified by quartiles according to the factor score in each pattern. Logistic regression was used to analyze the relationships between DV, DPs, and sarcopenia. The lowest quartile was defined as a reference, odds ratios (*ORs*), and 95% confidence intervals (*CIs*) were calculated in different models. Model 1 was a crude model; model 2 was adjusted by age, gender, and region; model 3 was adjusted by age, gender, region, body mass index (BMI), exercise activity, lifestyle, and total dietary energy; model 4 was adjusted by age, gender, region, BMI, exercise activity, lifestyle, total dietary energy, smoke status, and status of NCDs. All of the covariates were coded as dummy variables before the adjustment. Kruskal–Wallis rank test was used for the comparisons of DV score and nutrients intake among the highest quartiles of three DPs. The significance level of inter-group comparison was adjusted by the Bonferroni method. Dose-dependent relationships between sarcopenia and the percentage of energy from carbohydrate (PEC) and the percentage of energy from fat (PEF) were assessed by restricted cubic splines regression models. The models were adjusted for age, gender, region, BMI, exercise activity, lifestyle, smoke, status of NCDs, total dietary energy, protein amount per body weight, intake amount of dietary carbohydrate and fat, and DP score. All of the statistical analyses were performed using SAS 9.4 (SAS Institute, Cary, NC, USA) and R studio 3.1.3. *p*-value < 0.05 was considered as statistically significant.

## 3. Results

### 3.1. Particpants Characteristics

Participants characteristics of all of the 861 subjects were presented in Table 1. The prevalence rate of sarcopenia was 15.3% (132/861) according to the diagnosis criterion of AWGS2014. There were no significant differences among the subjects with or without sarcopenia, regarding gender, region, exercise activity, lifestyle, and the status of NCDs. While, subjects with sarcopenia, with lower level of BMI (*p-*value < 0.001), were significantly older than subjects without sarcopenia (*p-*value < 0.001). Current smokers (20.1%) showed significantly higher prevalence of sarcopenia than non-smokers (13.9%).

### 3.2. Consumption Correlations among Food Groups

**Figure 1:**
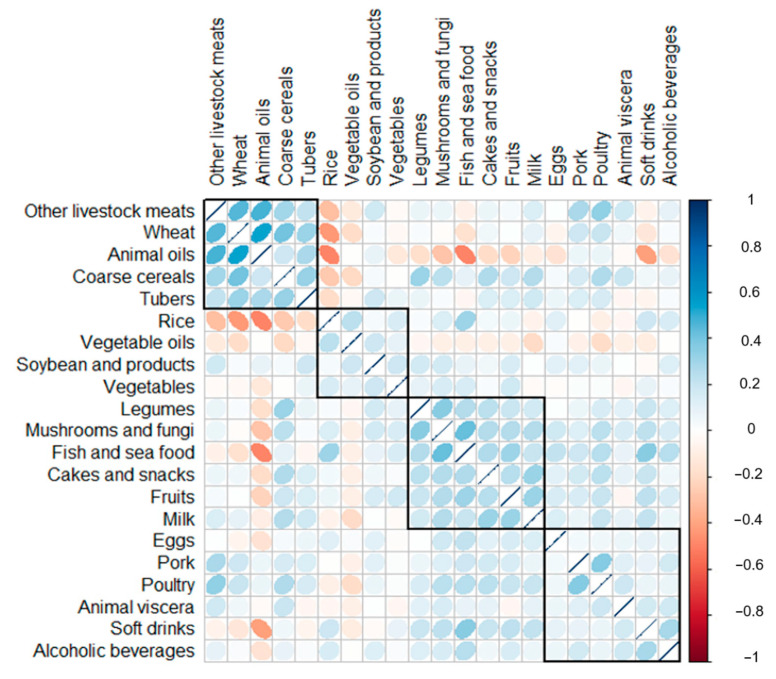
Consumption correlations among different food groups.

Consumption correlations among all of the 21 food groups were presented by correlation matrix in Figure 1. According to the order of hierarchical clustering, four identical clusters were identified and ranked. Other livestock meats (beef, mutton, and the other red meats except for pork), wheat, animal oils, coarse cereals, and tubers showed the most intensive correlations and ranked as the first cluster. Rice, vegetable oils, soybean and its products, and vegetables were identified as the second cluster. Legumes, mushrooms and fungi, fish and seafood, cakes and snacks, fruits, and milk were ranked as the third food cluster. The other food groups such as eggs, pork, poultry, animal viscera, soft drink, and alcoholic beverages combined into the fourth cluster.

### 3.3. Three Identified DPs in Older Subjects

Three DPs were identified in the present study among all of the 861 subjects (Table 2). Twenty-one food group were ranked and presented as four food cluster identified in Figure 1. According to the factor loadings of all of the 21 food groups, three DPs were identified and named as “cereals-tubers-animal oils” pattern, “mushrooms–fruits–milk” pattern and “animal foods” pattern, respectively. The “cereals–tubers–animal oils” pattern explained 13.8% variance of all of the food groups among all of the older subjects, the “mushrooms–fruits–milk” pattern explained 10%, and the “animal foods” pattern explained 7%, all of the three patterns explained more than 30% variance of food intake in this study.

### 3.4. Partical Correlations between DV Score, DPs Score, and Anthropometric Characteristics

Partial correlations between DV, DPs, and anthropometric characteristics related to muscle health were shown in Table 3. With the adjustment of age, gender, and region, FFM, BMC, handgrip strength, and gait speed were positively correlated with DV scores. For the DP1, compared with WC (*r* = 0.09, *p-*value = 0.007) and gait speed (*r* = 0.07, *p-*value = 0.032), scores of DP1 showed higher partial correlation coefficients with PBF (*r* = 0.21, *p-*value < 0.001) and VFA (*r* = 0.20, *p-*value < 0.001). For the DP2, significant correlations were observed between the DP2 score and CC (*r* = 0.12, *p-*value < 0.001), FFM (*r* = 0.12, *p-*value <0.001), BMC (*r* = 0.12, *p-*value < 0.001), grip strength (*r* = 0.07, *p-*value = 0.044), gait speed (*r* = 0.09, *p-*value = 0.009), and SMI (*r* = 0.11, *p-*value = 0.002), respectively. For the DP3, only significant correlations were observed between DP3 score and grip strength (*r* = 0.08, *p-*value = 0.015).

### 3.5. Associations between DV, DPs, and Sarcopenia

The associations between DV, DPs, and sarcopenia were shown in Table 4. Compared with the lowest quartile of DV score, higher quartiles of DV were negatively associated with sarcopenia. While the significant associations were attenuated after multiple adjustments. In the adjusted model, the second quartile (*OR* = 0.51, *95% CI* = 0.28~0.96) and the third quartile showed negative associations with sarcopenia (*OR* = 0.35, *95% CI* = 0.16~0.75). For DPs, the DP1 and the DP3 showed no significant association with sarcopenia defined by AWGS2014. However, compared with the lowest quartile of DP2, the increase of pattern score reduced the *OR* values of sarcopenia in the crude model and the adjusted model. In the adjusted model, compared with the lowest quartile, the highest quartile of DP2 score showed strongly negative association with sarcopenia defined by AWGS2014 (*OR* = 0.33, *95% CI* = 0.14~0.77, *p*-trend = 0.009).

### 3.6. Different DV Score and Nutrients Intake of Three Identified DPs

Table 5 presented the differences in general characteristics, prevalence of sarcopenia, food variety, and relevant nutrient intake among the subjects from the highest quartiles of DPs. Characteristics and prevalence of sarcopenia among different quartiles of DPs have been shown in Tables S1–S3, respectively. The lowest prevalence rate of sarcopenia was found in the highest quartile of DP2 (8.4%). Compared with the other two patterns, DP2 scored higher in the food variety (13.4 ± 2.2) and protein variety (3.3 ± 1.2). Meanwhile, the highest quartile of DP1 scored only 10.3 ± 3.3 at overall food variety and 2.0 ± 1.3 at protein source variety, both of them were the lowest among all of the three patterns (*p-*value < 0.001). Besides the differences of food variety, three DPs showed more differences regarding the intake amounts and compositions of macronutrients. Compared with the other patterns, DP2 showed the highest intake amounts of dietary carbohydrates (324.7 g) per day. There was no significant difference in total protein intake between DP2 and DP3. In line with the intake amounts of macronutrients, DP2 showed the highest percentage of energy supply from carbohydrate (53.4%) and the lowest energy from dietary fat (31.2%). The highest intake amounts of minerals related to bone and muscle health, such as calcium, phosphorus, magnesium, potassium, were all observed in DP2. However, there was no significant difference among the intake amounts of dietary branched-chain amino acids (BCAAs) between DP2 and DP3.

### 3.7. Associations between Sarcopenia and Dietary Energy Composition of Carbohydrate and Fat

**Figure 2:**
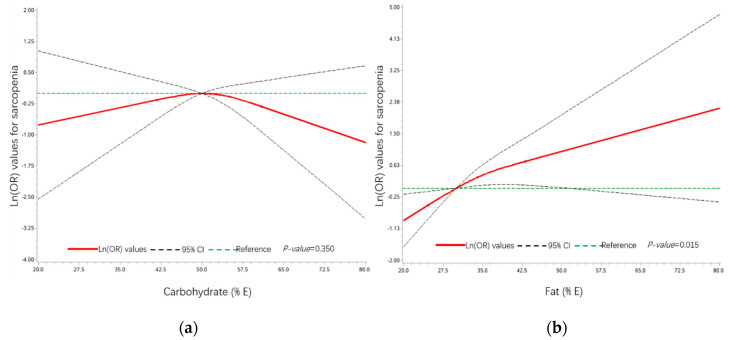
Associations between sarcopenia and dietary energy composition of carbohydrate and fat. (**a**) Non-linear association between sarcopenia and energy supply of carbohydrate and (**b**) non-linear association between sarcopenia and energy supply of fat. Models were adjusted by age, gender, region, BMI, exercise activity, lifestyle, dietary energy, smoke status, status of NCDs, protein amount per body weight, dietary consumption of carbohydrate and fat, and DP2 score.

Dose-dependent relationships between sarcopenia and dietary energy composition were assessed by restricted cubic splines regression (Figure 2). Models were adjusted by age, gender, region, BMI, exercise activity, lifestyle, smoke, status of NCDs, dietary energy, protein amount per body weight, dietary consumption of carbohydrate and fat, and DP2 score, which showed significant association with sarcopenia in the present study. As shown in Figure 2a, with the reference of 50% PEC level showed no significant association with and sarcopenia (*p-*value = 0.350). A significant association was observed between sarcopenia and PEF. With the reference of 30%, lower PEF (<30%) showed a significantly negative association with sarcopenia, 30~50% PEF showed significantly positive association with sarcopenia (*p-*value = 0.015), and higher PEF showed no significant association with sarcopenia in Figure 2b.

## 4. Discussion

With the increasing consideration for the whole diet, researchers developed DPs for the assessment of dietary quality [7]. While, to the best of our knowledge, several studies have been conducted to explore the association between DP and sarcopenia, but fewer of them compared the DV, dietary energy composition, and related nutrients among different DPs associated with sarcopenia [4]. In the present study, we assessed the DPs of 861 community-dwelling older people from three regions of China. “Cereals–tubers–animal oils” pattern, “mushrooms–fruits–milk” pattern, and “animal foods” pattern were progressively identified (Table 2). A higher dietary score in the “mushrooms–fruits–milk” pattern showed a lower risk of sarcopenia, the other two patterns showed no significant association with sarcopenia, using the recommended criterion of AWGS2014. Besides, similar trends were also observed between the three DPs, DV, and sarcopenia, using the criterion of AWGS2019 (Table S4). To explore the different associations between DPs and sarcopenia, we assessed the differences in DV, nutrients intake, and dietary energy composition among the three identified DPs. Compared with the other two DPs, subjects in the highest quartile of “mushrooms–fruits–milk” pattern showed higher score of DV, abundant intake of protein and relevant nutrients related to muscle health, and a significantly lower PEF. Furthermore, we found a non-linear dose-dependent association between sarcopenia and PEF. A lower PEF (<30%) was negatively associated with sarcopenia, and a higher PEF (30~50%) in dietary energy was positively associated with sarcopenia. Our study partially expounded the relationships between identified DPs and sarcopenia in community-dwelling older Chinese, and the potential mechanism regarding nutrients intake and dietary energy composition. Relevant results provided scientific evidence for the prevention and management of sarcopenia.

In 2015, a cross-sectional study in Iran identified three DPs from 300 middle-aged people from one district and discussed their relationship with sarcopenia. Only the Mediterranean pattern, characterized by higher consumption of vegetables, fish, fruits, and nuts, could reduce the risk of sarcopenia [34]. The other two patterns characterized by higher consumption of fast foods, sugar, and soy, or higher consumption of animal proteins, potatoes, and refined grains showed no significant association with sarcopenia. Chan et al. analyzed three DPs from Chinese community-dwelling older subjects in Hong Kong, China. After a 4-year observation, older males with a higher score in DQI-I or “vegetables–fruits” pattern, characterized by a higher loading of fruits and vegetables, showed a lower likelihood of sarcopenia only at the baseline [35]. Three DPs were identified from community-dwelling older people living in three different regions of China in the present study. The “mushrooms–fruits–milk” pattern, with higher factor loading of coarse cereals, mushrooms and fungi, fruits, milk, and fish, showed a higher score in DV and a lower risk of sarcopenia.

Nutrients related to sarcopenia have been compared among different DPs in the present study to explore the potential mechanism regarding DP and sarcopenia. Protein has been recognized as one of the most important nutrients in muscle health [36]. Some researchers recommended an abundant intake of dietary protein for the prevention and management of sarcopenia in older people [37,38]. However, the ideal intake amount of daily protein is still unclear. Morais et al. recommended an ideal protein intake range from 1.0 to 1.3 g/kg/d for nitrogen balance in healthy older people [39]. Some experts increased the protein intake amount from 0.83 to 1.2~1.6 g/kg/d for older people for anabolic resistance and muscle protein synthesis [40,41,42]. While, a recent study in the UK reported that, the “traditional British” dietary pattern, with a higher proportion of older people (over 85 years old) ate butter, red meats/meat dishes, gravy, potatoes, vegetables, and sweets, increased the risk of sarcopenia at baseline and after a 3-year follow-up in participants with good protein intake [43]. In our study, the protein intake amount in the highest quartile of subjects in the “mushrooms–fruits–milk” pattern reached 1.7 g/kg/d, and subjects in the highest quartile of the “animal foods” pattern were the same (Table 5). Relevant indicators of renal function were compared between subjects in different DPs. There was no significant difference of serum creatinine among the three DPs. Meanwhile, the mean level of glomerular filtration rate in DP2 was even higher than the other two patterns, which indicated a higher protein intake level (1.7 g/kg/d) in DP2 and DP3 may not increase the risk of renal failure among Chinese community-dwelling older people (Table S5). However, the “animal foods” pattern, with an abundant intake of dietary protein (1.7 g/kg/d), showed no significant association with sarcopenia. In line with the previous study [43], this result indicated other potential mechanisms should be also be mentioned and discussed, except for the intake amount of dietary protein.

Other micronutrients related to bone and muscle health were then compared among the three identified DPs (Table 5). With the highest score of DV, older subjects in the highest quartile of the “mushrooms–fruits–milk” pattern showed a higher intake of minerals (calcium, phosphorus, magnesium, and potassium) related to bone and muscle health. BCAAs were also analyzed in our study. Leucine, isoleucine, and valine, account for more than 50% of muscle protein essential amino acid [44]. A previous study reported that BCAAs intake (5.6 g) could increase the synthesis of muscle protein after physical training [45]. A meta-analysis including 16 studies and 999 subjects reported a mean effect of 0.99 kg increase in lean body mass after leucine supplementation in all of the participants, and 1.14 kg increase of lean body mass in the participants with manifested sarcopenia [46]. Very few studies have associated dietary BCAA with sarcopenia in community-dwelling people, and there is still little evidence for the dietary BCAA intake for the prevention of sarcopenia in older people. In the present study, two DPs were identified with a higher intake of BCAAs. The World Health Organization (WHO) recommended daily requirements of valine (26 mg/kg/d), leucine (39 mg/kg/d), and isoleucine (20 mg/kg/d) for older people in 2007 [47]. With an abundant intake of dietary protein (1.7 g/kg/d) in older people, all of the intake amounts of daily BCAAs in the highest quartiles of the “mushrooms–fruits–milk” pattern (valine 55.8 mg/kg/d, leucine 85.6 mg/kg/d, isoleucine 47.7 mg/kg/d) were higher than the recommendations of WHO, respectively. However, the “animal foods” pattern with the same protein intake (1.7 g/kg/d) and relevant higher intake level of BCAAs (valine 58.5 mg/kg/d, leucine 89.5 mg/kg/d, isoleucine 50.6 mg/kg/d) showed no significant association with sarcopenia in older people.

To further explore the different associations between two protein-sufficient DPs with sarcopenia, the dose-dependent relationship between sarcopenia and dietary fat intake was analyzed, which was one of the most significant differences between DP2 and DP3. There are several potential mechanisms regarding dietary fat and muscle mass decline in previous studies. Intermuscular adipocytes and intramyocellular lipids (IMCLs) caused by over intake of dietary fat were two of the most concerned underlying factors at present [48,49]. For intermuscular adipocytes, in vitro evidence suggested that adipocytes from visceral fat in obese could decrease the expression of contractile proteins such as troponin, titin, and myosin in myotubes, and consequently provoke dysfunction and atrophy in muscle cells [50]. As for IMCLs, plasma fatty acids (FAs) moved into skeletal muscle cells through FA transport proteins, such as a cluster of differentiation 36 (CD36), and then entered the oxidation process, triglyceride synthesis in muscle cells [49,51]. Lipid accumulation in skeletal muscle dramatically increased when FA uptake outpaced FA oxidation in muscle cell, and then generated intermuscular adipose tissue and IMCLs [49]. Lipid droplets (LDs) in muscle cells, covered with multiple perilipins, mainly composed by IMCLs, triglyceride and sterol esters, played a vital role in intracellular lipid homeostasis [52,53]. High-fat diet (HFD) increased the expression of CD36, and then increased FA uptake in skeletal muscle cells [54]. Long-term HFD feeding significantly increased the size of LDs and the expression of perilipins in skeletal muscle of mice [55]. Over-sized LDs progressively altered insulin signaling and increased the accumulation of oxidation intermediates, such as diacylglycerols and ceramides, which are implicated in lipotoxic effect on cells [56]. Oxidative stress and lipotoxicity could produce muscle atrophy and muscular weakness, and then lead to muscle mass decline and sarcopenia [57]. In recent years, insulin resistance caused by lipid infusion has been widely discussed as another risk factor of muscle decline [48]. Previous studies have demonstrated well that insulin could increase the transportation of amino acid into cells and stimulate muscle protein synthesis in skeletal muscle [48,58]. Meanwhile, insulin also played a clear role in reducing muscle protein breakdown in humans [59]. Those effects made insulin a vital role in the regulation of protein synthesis in muscle cell. Previous studies indicated that insulin resistance (IR) may cause muscle protein wasting by upregulating the phosphorylation of elongation factor 2α [60], activating the caspase-3 and the ubiquitin-proteasome proteolytic pathway in skeletal muscle cells [61]. The above studies partially explained the potential mechanism regarding dietary fat and sarcopenia, over intake of dietary fat may increase lipid infusion in intermuscular adipocytes, generate oxidative stress, lipotoxicity, and IR in skeletal muscle cells, and then lead to protein wasting and autophagy in muscle cells, and finally cause skeletal muscle mass decline and sarcopenia. Nevertheless, the reasonable intake amount of dietary fat for the prevention of sarcopenia is still unclear. Partially in line with those previous studies, our analysis observed a lower fat diet (dietary PEF < 30%) showed a significantly negative association with sarcopenia, and a higher fat diet (dietary PEF ranged from 30~50% showed a significantly positive association with sarcopenia (Figure 2). The result provided a reasonable range of dietary fat consumption for the prevention of sarcopenia, and may partially explain the non-significant association between the “animal foods” pattern and sarcopenia. Though this kind of DP has abundant protein intake, its higher PBF in daily diet may increase the risk of sarcopenia in older people. Besides the intake amount, several studies reported the nature of dietary fat was also important for skeletal muscle. Palmitic acid, a kind of saturated fatty acid, induced inflammation and IR in skeletal muscle cells, and has been used for IR modeling [62,63]. An in vivo study reported that diet enriched in oleate restored muscle protein synthesis and improve insulin sensitivity in old rats, while the diet enriched in palmitate showed adverse effects [64]. Oleate could also relieve the adverse effects caused by palmitate though promoting triglyceride accumulation and mitochondrial beta-oxidation in muscle cells [65]. However, there is still a limited amount of relevant evidence. More studies need to be conducted to discuss the relationship between intake amount of dietary fat, fat composition, and sarcopenia.

There are some limitations in the present study. The present study was a cross-sectional study, more cohort and intervention studies are required to explore the relationship between diet and sarcopenia in the future. Furthermore, there are no uniform diagnosis criteria of sarcopenia at present, AWGS 2014 was chosen as the standard criteria of sarcopenia in the present study.

## 5. Conclusions

Three DPs, “cereals–tubers–animal oils” pattern, “mushrooms–fruits–milk” pattern, and “animal foods” pattern, were identified from community-dwelling older people in three regions of China. Only the “mushrooms–fruits–milk” pattern was negatively associated with sarcopenia. Compared with the other two DPs, the “mushrooms–fruits–milk” pattern showed abundant protein intake and lower dietary PEF, indicating that protein and fat may both involved in the development of sarcopenia and might play different roles. In the present study, the dose-dependent relationship between PEF and sarcopenia was discussed for the first time. Low PEF (<30%) was negatively associated with sarcopenia. High PEF (30%~50%) was positively associated with sarcopenia. Therefore, besides protein, dietary fat and PFE may also be considered in the prevention and management of sarcopenia.

## Figures and Tables

**Table 1:** Participants characteristics.

Characteristic	Total	Sarcopenia		
		Yes	No	*p-*Value
**Subjects**	861 (100.0)	132 (15.3)	729 (84.7)	
Age (y)	71.0 ± 4.8	74.2 ± 5.4	70.4 ± 4.4	<0.001
BMI (kg/m^2^)	23.8 ± 3.6	21.2 ± 3.5	24.3 ± 3.4	<0.001
**Gender**				
Male	405 (47.0)	62 (15.3)	343 (84.7)	0.986
Female	456 (53.0)	70 (15.4)	386 (84.6)	
**Region**				
South China (Yuexiu)	286 (33.2)	35 (12.2)	251 (87.8)	0.092
Middle China (Taicang)	311 (36.1)	47 (15.1)	264 (84.9)	
North China (Wuyuan)	264 (30.7)	50 (18.9)	214 (81.1)	
**Exercise activity**				
Low	20 (2.3)	5 (25.0)	15 (75.0)	0.257
Moderate	485 (56.3)	79 (16.3)	406 (83.7)	
Heavy	356 (41.4)	48 (13.5)	308 (86.5)	
**Lifestyle**				
Living alone	87 (10.1)	13 (14.9)	74 (85.1)	0.401
Living with spouse	656 (76.2)	96 (14.6)	560 (85.4)	
Living with others	118 (13.7)	23 (19.5)	95 (80.5)	
**Current smoker**				
Yes	204 (23.7)	41 (20.1)	163 (79.9)	0.031
No	657 (76.3)	91 (13.9)	566 (86.2)	
**NCDs**				
Hypertension	417 (48.4)	63 (15.1)	354 (84.9)	0.86
T2D	113 (13.1)	13 (11.5)	100 (88.5)	0.226
CVD	178 (20.7)	31 (17.4)	147 (82.6)	0.386

Quantitative data are shown as mean ± SD, categorical data are shown as *n* (%). Abbreviations: BMI—Body mass index; NCDs—Non-communicable chronic diseases; T2D—Type 2 diabetes; CVD—Cardiovascular disease.

**Table 2:** Food group factor loadings for different dietary patterns (DPs).

Cluster	Food Group	DP1	DP2	DP3
Cluster 1	Other livestock meats	0.351	−0.013	0.689
	Wheat	0.653	−0.036	0.240
	Animal oils	0.583	−0.373	0.129
	Coarse cereals	0.442	0.416	0.069
	Tubers	0.550	0.149	0.056
Cluster 2	Rice	−0.714	0.120	0.031
	Vegetable oils	−0.351	−0.144	−0.015
	Soybean and products	−0.016	0.309	0.182
	Vegetables	−0.111	0.173	−0.059
Cluster 3	Legumes	0.064	0.353	0.105
	Mushrooms and fungi	0.141	0.596	0.104
	Fish and seafood	−0.244	0.428	0.235
	Cakes and snacks	0.183	0.512	0.055
	Fruits	−0.023	0.553	0.050
	Milk	0.243	0.583	−0.049
Cluster 4	Eggs	−0.026	0.216	0.194
	Pork	−0.031	0.112	0.697
	Poultry	0.126	0.092	0.733
	Animal viscera	−0.053	0.019	0.380
	Soft drinks	−0.070	0.305	−0.058
	Alcoholic beverages	−0.319	−0.025	0.129
Variance explained (%)		13.8	10	7

Abbreviation: DP1—Cereals–tubers–animal oils pattern; DP2—Mushrooms–fruits–milk pattern; DP3—Animal foods pattern.

**Table 3:** Partial correlations between dietary variety (DV), DPs, and anthropometric characteristics.

Characteristic	DV Score		DP1 Score		DP2 Score		DP3 Score	
	*r*	*p-*Value	*r*	*p-*Value	*r*	*p-*Value	*r*	*p-*Value
MUAC	0.02	0.574	0.03	0.402	0.04	0.254	−0.03	0.4
CC	0.02	0.512	0.07	0.056	0.12	<0.001	−0.06	0.09
WC	−0.02	0.51	0.09	0.007	0.04	0.212	−0.03	0.34
PBF	−0.03	0.376	0.21	<0.001	0.02	0.588	−0.03	0.325
VFA	−0.02	0.619	0.20	<0.001	0.04	0.264	−0.03	0.333
FFM	0.07	0.037	0.06	0.061	0.12	<0.001	<0.01	0.99
BMC	0.08	0.016	0.02	0.511	0.12	<0.001	−0.02	0.62
Grip strength	0.08	0.014	0.05	0.128	0.07	0.044	0.08	0.015
Gait speed	0.08	0.014	0.07	0.032	0.09	0.009	0.05	0.169
SMI	0.03	0.426	0.06	0.075	0.11	0.002	−0.03	0.362

Partial correlations were obtained with the adjustment of age, gender, and region. Abbreviation: MUAC—Mid-upper arm circumference; CC—Calf circumference; WC—Waist circumference; PBF—Percentage of body fat mass; VFA—Visceral fat area; FFM—Fat free mass; BMC—Bone minerals content; SMI—Skeletal muscle index. DV—Dietary variety; DP1—“Cereals–tubers–animal oils” pattern; DP2—“Mushrooms–fruits–milk” pattern; DP3—“Animal foods” pattern.

**Table 4:** Associations between DV, DPs, and sarcopenia.

DP	Model 1		Model 2		Model 3		Model 4	
	OR 95%CI	*p*-Trend	OR 95%CI	*p*-Trend	OR 95%CI	*p*-Trend	OR 95%CI	*p*-Trend
**DV score**								
Q1 ^a^	Ref	0.008	Ref	0.071	Ref	0.059	Ref	0.099
Q2	0.64 (0.38, 1.07)		0.69 (0.40, 1.19)		0.51 (0.28, 0.95)		0.51 (0.28, 0.96)	
Q3	0.37 (0.20, 0.71)		0.41 (0.21, 0.81)		0.33 (0.15, 0.70)		0.35 (0.16, 0.75)	
Q4	0.50 (0.30, 0.84)		0.59 (0.33, 1.08)		0.49 (0.24, 0.97)		0.52 (0.26, 1.05)	
**DP1**								
Q1 ^b^	Ref	0.45	Ref	0.345	Ref	0.619	Ref	0.689
Q2	0.76 (0.44, 1.32)		0.67 (0.36, 1.22)		0.63 (0.31, 1.28)		0.63 (0.31, 1.27)	
Q3	1.18 (0.71, 1.96)		1.03 (0.45, 2.34)		1.28 (0.51, 3.17)		1.28 (0.51, 3.23)	
Q4	1.07 (0.64, 1.80)		0.68 (0.27, 1.76)		0.80 (0.28, 2.30)		0.85 (0.29, 2.47)	
**DP2**								
Q1 ^c^	Ref	<0.001	Ref	<0.001	Ref	0.006	Ref	0.009
Q2	0.64 (0.40, 1.04)		0.71 (0.41, 1.20)		0.82 (0.46, 1.48)		0.81 (0.45, 1.46)	
Q3	0.46 (0.28, 0.77)		0.47 (0.26, 0.85)		0.53 (0.27, 1.03)		0.53 (0.27, 1.04)	
Q4	0.29 (0.17, 0.52)		0.30 (0.15, 0.60)		0.32 (0.14, 0.75)		0.33 (0.14, 0.77)	
**DP3**								
Q1 ^d^	Ref	0.313	Ref	0.673	Ref	0.863	Ref	0.807
Q2	0.87 (0.52, 1.45)		0.81 (0.48, 1.38)		0.63 (0.34, 1.16)		0.63 (0.34, 1.17)	
Q3	0.87 (0.52, 1.46)		0.91 (0.53, 1.56)		0.79 (0.42, 1.47)		0.82 (0.43, 1.52)	
Q4	0.75 (0.44, 1.27)		0.85 (0.48, 1.52)		0.91 (0.44, 1.89)		0.87 (0.41, 1.82)	

Model 1: Crude model. Model 2: adjusted by age, gender, and region. Model 3: adjusted by age, gender, region, BMI, exercise activity, lifestyle, and total dietary energy. Model 4: adjusted by age, gender, region, BMI, exercise activity, lifestyle, total dietary energy, smoke status, status of NCDs. ^a^ DV score ranged from 3 to 20; Q1 (3~9), Q2 (10~13), Q3 (14~16), Q4 (17~20). ^b^ DP1 score ranged from −3.41 to 4.67; Q1 (−3.41~−0.75), Q2 (−0.74~−0.07), Q3 (−0.06~0.68), Q4 (0.69~4.67). ^c^ DP2 score ranged from −2.09 to 6.12; Q1 (−2.09~−0.66), Q2 (−0.65~−0.21), Q3 (−0.20~0.48), Q4 (0.49~6.12). ^d^ DP3 score ranged from −1.39 to 14.2; Q1 (−1.39~−0.57), Q2 (−0.56~−0.28), Q3 (−0.27~0.27), Q4 (0.28~14.2). Abbreviation: DV—Dietary variety; DP1—“Cereals–tubers–animal oils” pattern; DP2—“Mushrooms–fruits–milk” pattern; DP3—“Animal foods” pattern; BMI—Body-mass index.

**Table 5:** Different food variety and nutrients intake among the highest quartiles of three DPs.

Characteristic	DP1 (*n* = 215)	DP2 (*n* = 215)	DP3 (*n* = 215)	*p-*Value
**General** **characteristic**				
Age (y)	71.3 ± 5.1	70.4 ± 4.6	69.9 ± 4.2 ^a^	0.025
BMI (kg/m^2^)	23.9 ± 4.0	24.1 ± 3.2	23.6 ± 3.3	0.318
Male (*n*, %)	104 (48.4)	100 (46.5)	138 (64.2) ^ab^	<0.001
Sarcopenia (*n*, %)	35 (16.3)	18 (8.4) ^a^	29 (13.5)	0.044
**Food variety score of DQI-I**				
Overall food variety score	10.3 ± 3.3	13.4 ± 2.2 ^a^	11.2 ± 2.8 ^ab^	<0.001
Protein variety score	2.0 ± 1.3	3.3 ± 1.2 ^a^	2.7 ± 1.3 ^ab^	<0.001
Total variety score	12.7 ± 4.6	17.5 ± 3.2 ^a^	14.4 ± 3.9 ^ab^	<0.001
**Dietary nutrients intake and composition**				
Energy (kcal)	2190.8 ± 855.2	2468.4 ± 788.5 ^a^	2555.1 ± 776.8 ^a^	<0.001
Carbohydrate (g)	272.9 ± 121.0	324.7 ± 106.6 ^a^	283.7 ± 102.6 ^b^	<0.001
Protein (g)	77.6 ± 42.7	101.5 ± 39.3 ^a^	102.1 ± 42.1 ^a^	<0.001
Protein per weight (g/kg)	1.3 ± 0.7	1.7 ± 0.7 ^a^	1.7 ± 0.7 ^a^	<0.001
Fat (g)	89.6 ± 46.3	87.1 ± 41.5	108.5 ± 44.6 ^ab^	<0.001
PEC (%E)	49.9 ± 11.8	53.4 ± 9.5 ^a^	44.6 ± 10.0 ^ab^	<0.001
PEP (%E)	13.7 ± 2.8	16.4 ± 2.9 ^a^	16.0 ± 3.6 ^a^	<0.001
PEF (%E)	37.2 ± 12.1	31.2 ± 8.6 ^a^	38.3 ± 10.3 ^b^	<0.001
Calcium (mg)	553.2 ± 379.0	871.1 ± 373.8 ^a^	620.9 ± 343.6 ^ab^	<0.001
Phosphorus (mg)	1246.9 ± 643.6	1624.6 ± 575.9 ^a^	1468.6 ± 592.0 ^ab^	<0.001
Magnesium (mg)	372.8 ± 204.5	483.5 ± 186.9 ^a^	409.8 ± 178.8 ^ab^	<0.001
Potassium (mg)	2418.9 ± 1317.3	3368.3 ± 1242.1 ^a^	2790.8 ± 1245.1 ^ab^	<0.001
Valine (mg/kg)	44.6 ± 27.0	55.8 ± 30.1 ^a^	58.5 ± 30.5 ^a^	<0.001
Leucine (mg/kg)	68.6 ± 42.9	85.6 ± 47.3 ^a^	89.5 ± 47.9 ^a^	<0.001
Isoleucine (mg/kg)	37.5 ± 23.7	47.7 ± 26.6 ^a^	50.6 ± 27.2 ^a^	<0.001

^a^ Statistically different with DP1; ^b^ Statistically different with DP2. Abbreviation: DP1—“Cereals–tubers–animal oils” pattern; DP2—“Mushrooms–fruits–milk” pattern; DP3—“Animal foods” pattern; BMI—Body mass index; PEC—Percentage of energy from carbohydrate; PEP—Percentage of energy from protein; PEF—Percentage of energy from fat.

## Supplementary Materials

The following are available online at https://www.mdpi.com/2072-6643/12/12/3689/s1, Table S1: Participants characteristics among different quartiles of DP1, Table S2: Participants characteristics among different quartiles of DP2, Table S3: Participants characteristics among different quartiles of DP3, Table S4: Associations between dietary variety, DPs and sarcopenia defined by AWGS2019, Table S5: Renal indicators among the highest quartiles of three DPs.
