# Supplementary material for: Dietary Pattern and Dietary Energy from Fat Associated with Sarcopenia in Community-Dwelling Older Chinese People: A Cross-Sectional Study in Three Regions of China
Source: Nutrients. 2020 Nov 30;12(12):3689. doi: 10.3390/nu12123689 (PMC7760936; doi:10.3390/nu12123689)
Supplement: Supplementary file 1 [file nutrients-12-03689-s001.pdf]

Supplementary Table S1. Participants characteristics among different quartiles of DP1.

Characteristic	Q1	Q2	Q3	Q4	P-value
**Subjects**	215 (25.0%)	215 (25.0%)	216 (25.0%)	215 (25.0%)	
Age (y)	70.5±4.0	71.5±4.7	70.7±5.1	71.3±5.1	0.050
BMI (kg/m2)	23.4±3.1	23.4±3.2	24.6±3.8	23.9±4.0	0.002
**Gender**					
Male	129 (60.0)	89 (41.4)	83 (38.4)	104 (48.4)	<0.001
Female	86 (40.0)	126 (58.6)	133 (61.6)	111 (51.6)	
**Region**					
South China (Yuexiu)	30 (14.0)	92 (42.8)	125 (57.9)	39 (18.1)	
Middle China (Taicang)	183 (85.1)	120 (55.8)	8 (3.7)	0 (0)	<0.001
North China (Wuyuan)	2 (0.9)	3 (1.4)	83 (38.4)	176 (81.9)	
**Exercise activity**					
Low	7 (3.2)	6 (2.8)	4 (1.9)	3 (1.4)	
Moderate	116 (54.0)	143 (66.5)	133 (61.6)	93 (43.2)	<0.001
Heavy	92 (42.8)	66 (30.7)	79 (36.5)	119 (55.4)	
**Lifestyle**					
Living alone	13 (6.1)	31 (14.4)	20 (9.2)	23 (10.7)	
Living with spouse	173 (80.5)	152 (70.7)	165 (76.4)	166 (77.2)	0.134
Living with others	29 (13.4)	32 (14.9)	31 (14.4)	26 (12.1)	
**Current smoker**					
Yes	59 (27.4)	34 (15.8)	47 (21.8)	64 (29.8)	0.003
No	156 (72.6)	181 (84.2)	169 (78.2)	151 (70.2)	
**NCDs**					
Hypertension	102 (47.4)	126 (58.6)	103 (47.7)	86 (40.0)	0.002
T2D	18 (8.4)	30 (14.0)	45 (20.8)	20 (9.3)	<0.001
CVD	28 (13.0)	37 (17.2)	48 (22.2)	65 (30.2)	
**Sarcopenia**					
Yes	33 (15.4)	26 (12.1)	38 (17.6)	35 (16.3)	0.434
No	182 (84.7)	189 (87.9)	178 (82.4)	180 (83.7)	

Quantitative data are shown as mean±SD, categorical data are shown as n(%).DP1 score ranged from -3.41 to 4.67; Q1 (-3.41~-0.75), Q2 (-0.74~-0.07), Q3 (-0.06~0.68), Q4 (0.69~4.67). Abbreviations: NCDs-Non-communicable chronic diseases; T2D-Type 2 diabetes; CVD-Cardiovascular disease; DP1-"Cereals-tubers-animal oils" pattern.

Supplementary Table S2. Participants characteristics among different quartiles of DP2.

Characteristic	Q1	Q2	Q3	Q4	P-value

**Subjects**	215 (25.0%)	216 (25.0%)	215 (25.0%)	215 (25.0%)	
Age (y)	71.8±5.1	70.9±4.8	70.9±4.5	70.4±4.6	0.051
BMI (kg/m2)	23.5±4.1	23.8±3.4	24.0±3.5	24.1±3.2	0.312
**Gender**					
Male	98 (45.6)	108 (50.0)	99 (46.1)	100 (46.5)	0.789
Female	117 (54.4)	108 (50.0)	116 (54.0)	115 (53.5)	
**Region**					
South China (Yuexiu)	15 (7.0)	32 (14.8)	87 (40.5)	152 (70.7)	
Middle China (Taicang)	52 (24.2)	128 (59.3)	91 (42.3)	40 (18.6)	<0.001
North China (Wuyuan)	148 (68.8)	56 (25.9)	37 (17.2)	23 (10.7)	
**Exercise activity**					
Low	6 (2.8)	6 (2.8)	4 (1.9)	4 (1.9)	
Moderate	117 (54.4)	119 (55.1)	117 (54.4)	132 (61.4)	0.725
Heavy	92 (42.8)	91 (42.1)	94 (43.7)	79 (36.7)	
**Lifestyle**					
Living alone	24 (11.2)	26 (12.0)	21 (9.8)	16 (7.4)	
Living with spouse	169 (78.6)	157 (72.7)	168 (78.1)	162 (75.4)	0.251
Living with others	22 (10.2)	33 (15.3)	26 (12.1)	37 (17.2)	
**Current smoker**					
Yes	70 (32.6)	61 (28.2)	47 (21.9)	26 (12.1)	<0.001
No	145 (67.4)	155 (71.8)	168 (78.1)	189 (87.9)	
**NCDs**					
Hypertension	86 (40.0)	113 (52.3)	106 (49.3)	112 (52.1)	0.034
T2D	26 (12.1)	30 (13.9)	27 (12.6)	30 (14.0)	0.919
CVD	55 (25.6)	50 (23.2)	39 (18.1)	34 (15.8)	0.048
**Sarcopenia**					
Yes	51 (23.7)	36 (16.7)	27 (12.6)	18 (8.4)	<0.001
No	164 (76.3)	180 (83.3)	188 (87.4)	197 (91.6)	

Quantitative data are shown as mean±SD, categorical data are shown as n(%).DP2 score ranged from -2.09 to 6.12; Q1 (-2.09~-0.66), Q2 (-0.65~-0.21), Q3 (-0.20~0.48), Q4 (0.49~6.12). Abbreviations: NCDs-Non-communicable chronic diseases; T2D-Type 2 diabetes; CVD-Cardiovascular disease; DP2-"Mushrooms-fruits-milk" pattern.

Supplementary Table S3. Participants characteristics among different quartiles of DP3.

Characteristic	Q1	Q2	Q3	Q4	P-value
**Subjects**	215 (25.0%)	216 (25.0%)	215 (25.0%)	215 (25.0%)	
Age (y)	71.4±5.0	71.9±4.8	70.7±4.7	69.9±4.2	<0.001
BMI (kg/m2)	24.3±3.6	23.7±3.8	23.8±3.6	23.6±3.3	0.142
**Gender**					

Male	70 (32.6)	93 (43.1)	104 (48.4)	138 (64.2)	<0.001
Female	145 (67.4)	123 (56.9)	111 (51.6)	77 (35.8)	
**Region**					
South China (Yuexiu)	68 (31.6)	67 (31.0)	64 (29.8)	87 (40.5)	
Middle China (Taicang)	96 (44.7)	98 (45.4)	74 (34.4)	43 (20.0)	<0.001
North China (Wuyuan)	51 (23.7)	51 (23.6)	77 (35.8)	85 (39.5)	
**Exercise activity**					
Low	4 (1.9)	7 (3.2)	7 (3.3)	2 (0.9)	
Moderate	140 (65.1)	133 (61.6)	106 (49.3)	106 (49.3)	0.001
Heavy	71 (33.0)	76 (35.2)	102 (47.4)	107 (49.8)	
**Lifestyle**					
Living alone	30 (13.9)	22 (10.2)	14 (6.5)	21 (9.8)	
Living with spouse	159 (74.0)	154 (71.3)	173 (80.5)	170 (79.0)	0.049
Living with others	26 (12.1)	40 (18.5)	28 (13.0)	24 (11.2)	
**Current smoker**					
Yes	34 (15.8)	42 (19.4)	48 (22.3)	80 (37.2)	<0.001
No	181 (84.2)	174 (80.6)	167 (77.7)	135 (62.8)	
**NCDs**					
Hypertension	112 (52.1)	116 (53.7)	99 (46.1)	90 (41.9)	0.051
T2D	41 (19.1)	31 (14.4)	21 (9.8)	20 (9.3)	0.474
CVD	55 (25.6)	45 (20.8)	33 (15.4)	45 (20.9)	0.076
**Sarcopenia**					
Yes	37 (17.2)	33 (15.3)	33 (15.4)	29 (13.5)	0.766
No	178 (82.8)	183 (84.7)	182 (84.6)	186 (86.5)	

Quantitative data are shown as mean±SD, categorical data are shown as n(%). DP3 score ranged from -1.39 to 14.2; Q1 (-1.39~-0.57), Q2 (-0.56~-0.28), Q3 (-0.27~0.27), Q4 (0.28~14.2). Abbreviations: NCDs-Non-communicable chronic diseases; T2D-Type 2 diabetes; CVD-Cardiovascular disease; DP3-"Animal foods" pattern.

Supplementary Table S4. Associations between dietary variety, DPs and sarcopenia defined by AWGS2019.

DP	Model 1		Model 2		Model 3		Model 4	
	OR 95%CI	*P-trend*	*OR 95%CI*	*P-trend*	*OR 95%CI*	*P-trend*	*OR 95%CI*	*P-trend*
				DV score				
Q1	Ref	0.047	Ref	0.081	Ref	0.055	Ref	0.076
Q2	0.66 (0.40, 1.07)		0.68 (0.41, 1.13)		0.51 (0.29, 0.92)		0.51 (0.28, 0.92)	
Q3	0.54 (0.31, 0.94)		0.56 (0.31, 1.01)		0.46 (0.23, 0.90)		0.47 (0.24, 0.93)	
Q4	0.58 (0.36, 0.94)		0.59 (0.34, 1.03)		0.47 (0.25. 0.89)		0.49 (0.25, 0.93)	
				DP1				
Q1	Ref	0.68	Ref	0.394	Ref	0.783	Ref	0.882
Q2	1.00 (0.62, 1.62)		0.84 (0.49, 1.43)		0.86 (0.46, 1.60)		0.85 (0.46, 1.59)	
Q3	1.15 (0.72, 1.84)		0.91 (0.45, 1.84)		1.22 (0.55, 2.71)		1.25 (0.56, 2.78)	
Q4	1.06 (0.66, 1.71)		0.70 (0.31, 1.59)		0.86 (0.35, 2.15)		0.92 (0.36, 2.32)	
				DP2				
Q1	Ref	<0.001	Ref	<0.001	Ref	0.005	Ref	0.006
Q2	0.65 (0.41, 1.01)		0.65 (0.39, 1.06)		0.74 (0.43, 1.30)		0.74 (0.42, 1.28)	
Q3	0.49 (0.31, 0.78)		0.43 (0.25, 0.74)		0.48 (0.26, 0.90)		0.49 (0.26, 0.90)	
Q4	0.43 (0.26, 0.69)		0.35 (0.19, 0.64)		0.37 (0.18, 0.78)		0.37 (0.17, 0.79)	
				DP3				
Q1	Ref	0.903	Ref	0.364	Ref	0.264	Ref	0.321
Q2	0.91 (0.56, 1.47)		0.87 (0.53, 1.42)		0.69 (0.39, 1.23)		0.69 (0.38, 1.22)	
Q3	1.09 (0.68, 1.74)		1.20 (0.73, 1.95)		1.12 (0.63, 2.00)		1.13 (0.63, 2.02)	
Q4	0.97 (0.60, 1.56)		1.16 (0.69, 1.96)		1.30 (0.67, 2.52)		1.24 (0.63, 2.42)	

Model 1: Crude model. Model 2: adjusted by age, gender and region. Model 3: adjusted by age, gender, region, BMI, exercise activity, lifestyle, and total dietary energy. Model 4: adjusted by age, gender, region, BMI, exercise activity, lifestyle, total dietary energy, smoke status, status of NCDs. [a] DV score ranged from 3 to 20; Q1 (3~9), Q2 (10~13), Q3 (14~16), Q4 (17~20). [b] DP1 score ranged from -3.41 to 4.67; Q1 (-3.41~-0.75), Q2 (-0.74~-0.07), Q3 (-0.06~0.68), Q4 (0.69~4.67). [c] DP2 score ranged from -2.09 to 6.12; Q1 (-2.09~-

0.66), Q2 (-0.65~-0.21), Q3 (-0.20~0.48), Q4 (0.49~6.12). [d] DP3 score ranged from -1.39 to 14.2; Q1 (-1.39~-0.57), Q2 (-0.56~-0.28), Q3 (-0.27~0.27), Q4 (0.28~14.2). Abbreviation: DV-Dietary variety; DP1-"Cereals-tubers-animal oils" pattern; DP2-"Mushrooms-fruits-milk" pattern; DP3-"Animal foods" pattern.

Supplemental Table 5. Renal indicators among the highest quartiles of three DPs.

Characteristic	DP1 (n=215)	DP2 (n=215)	DP3 (n=215)	*P-value*
Urine acid (μmol/l)	316.9±87.8	365.0±96.8[a]	346.5±97.2[a]	<0.001
BUN (mmol/l)	7.0±2.0	6.3±1.5[a]	6.4±1.5[a]	<0.001
Scr (mg/dl)	0.9±0.2	0.9±0.2	0.9±0.2	0.139
Scys (mg/l)	1.2±0.3	1.0±0.2[a]	1.1±0.2[ab]	<0.001
eGFR (ml/min/1.73m2)	68.2±14.2	73.9±13.5[a]	73.0±13.3[a]	<0.001

eGFR was calculated based on the equation of CKD-EPI 2012. [a] Statistically different with DP1; [b] Statistically different with DP2. Abbreviation: BUN-Blood urea nitrogen; Scr-Serum creatine; Scys-Serum cystatin C; eGFR-estimated glomerular filtration rate.
